# Malperfusion and branch compromise in acute type A aortic syndrome

**DOI:** 10.1186/cc10797

**Published:** 2012-03-20

**Authors:** R Gomez Lopez, P Fernandez Ugidos, P Vidal Cortes, J Lopez Perez, J Priego Sanz, M Bouza Vieiro, A Aller Fernandez, L Seoane Quiroga, S Fojon Polanco

**Affiliations:** 1 Complexo Hospitalario Universitario de Ourense, Spain; 2 Complexo Hospitalario Universitario de A Coruña, Spain

## Introduction

Malperfusion is a factor associated with higher risk of death and complications in patients with acute type A aortic syndrome (AAAS). Our objective is to determine the incidence and characteristics of this disease in our population and to verify the relevance in morbidity and in-hospital mortality.

## Methods

A historical cohort study that includes all patients with AAAS admitted to the ICU after surgical management in a single institution from January 2000 to July 2010. Anatomical, clinical, biochemical, electrocardiographic and echocardiographic signs of ischemia were considered. The events of interest were death or major complication (neurological damage, multiorgan failure (MOF), acute lung injury (ALI), postoperative hemorrhage) during hospitalization.

## Results

A total of 65 patients were identified (24.6% women, 61.86 ± 12 years old, APACHE II score 12.9 ± 7.2, EuroSCORE 7.4 ± 2.6). Thirty-three (50.8%) presented branch compromise, affecting coronary arteries in 12 patients (18.4%) (symptomatic (S) seven (10.5%), asymptomatic (A) five (7.7%)), nine (13.8%) carotid (S five (7.7%), A four (6.1%)), 28 (43%) brachiocephalic or subclavian (S 17 (26.1%), A 11 (16.9%)), 15 (22.8%) mesenteric (S seven (10.5%), A eight (12.3%)), 13 (20%) renal (S nine (13.8%), A four (6.1%)), and 31 (47.7%) iliac (S 16 (24.6%), A 15 (23%)). Twenty-eight (43.1%) showed clinical ischemia of at least one system and 54 (83%) clinical signs of global hypoperfusion. Comparing patients with and without data of hypoperfusion, differences in incidence of death (45.5% vs. 18.8%, *P = *0.03), neurological complication (35.7% vs. 10.8%, *P = *0.03), MOF (16.6% vs. 25%. *P = *0.07) and ALI (21.3% vs. 29.6%, *P = *0.09) were found.

## Conclusion

More than 80% of the patients with AAAS suffered malper-fusion in our series. They had a higher risk of death and neurological complication during hospitalization.

